# Clinical Tools for Assessing One-Handed Skills in Children With Cerebral Palsy: An Umbrella Review

**DOI:** 10.1155/oti/8847527

**Published:** 2025-06-23

**Authors:** Samira Boroumand, Marzieh Pashmdarfard, Dorsa Hamedi, Afsoon Hassani Mehraban

**Affiliations:** ^1^Rehabilitation Research Center, Department of Occupational Therapy, School of Rehabilitation Sciences, Iran University of Medical Sciences, Tehran, Iran; ^2^Department of Occupational Therapy, School of Rehabilitation Sciences, Shahid Beheshti University of Medical Sciences, Tehran, Iran

**Keywords:** cerebral palsy, reliability, tool, unilateral, validity

## Abstract

**Introduction:** This study is aimed at identifying suitable tools capable of evaluating one-handed skills in children with cerebral palsy (CP).

**Methods:** In this article, the systematic reviews on upper extremity assessment tools for children with CP from 2000 to 2024 were identified in databases, including Google Scholar, PubMed, Web of Knowledge, and Scopus. Then, the obtained tools were evaluated, among which only those capable of assessing one-handed skills in children aged 3 years and older in accordance with the activity level of the International Classification of Functioning, Disability, and Health were selected to evaluate the quality of evidence and psychometric properties in children with CP using CanChild Outcome Measure Rating Form.

**Results:** A total of 13 systematic reviews were selected for further analysis. Subsequently, 149 tools were identified for initial evaluation, of which 18 were capable of assessing one-handed skills in children with CP. Among these, COPM, ACHES, and PMAL_R demonstrated excellent evidence for overall clinical utility.

**Conclusion:** This study showed that among the numerous tools available for assessing one-handed skills in children with CP, only a limited number have excellent evidence for overall clinical utility. These findings can serve as a basis for selecting more precise, evidence-based tools in the assessment, and rehabilitation interventions for these children.

## 1. Introduction

Cerebral palsy is the most common cause of physical disability in the early years of childhood [1]. Due to the many sensory, motor, and cognitive abnormalities that this disorder causes, it makes it difficult or sometimes impossible for children to perform many motor skills of the lower and upper extremities [2]. Studies have shown that 60%–80% of children with cerebral palsy suffer from upper extremity disorders [3]. Poor fine motor skills, reduced strength, and dexterity are some problems linked to the upper extremities of children with cerebral palsy, which are observed in one or two limbs based on the type of cerebral palsy [3, 4]. Upper extremity disorders make it difficult for children to independently perform many daily activities at home and school and even affect them while playing with their peers [5]. Therefore, various studies have always highlighted the need for the rehabilitation of the upper extremities of children with cerebral palsy and have emphasized the significance of designing a treatment program to improve the upper extremity function [2].

One of the main and undeniable components of designing a suitable treatment plan is the selection of accurate and appropriate assessment tools, which have been validated for target population based on age and diagnosis [6, 7]. Accurate assessment tools and appropriate psychometric features enable therapists to measure the patients' performance, thereby making it possible to make a correct judgment and design a suitable treatment plan. Also, these tools can help to detect the changes in patients over time, thereby making it possible to judge the effectiveness of therapeutic interventions [8].

Assessment tools can be divided in different ways [8]. Based on the International Classification of Functioning, Disability, and Health (ICF), tools can examine changes at the level of Body function and structure, activity and participation, and environment [9]. Accordingly, at the activity level, upper extremity tools examine individuals' ability to perform tasks or actions, including grasping, releasing, moving, manipulating objects, and using fine movements in the upper extremities. These tools can measure a person's functioning capacity in standard conditions or their actual daily life functioning [7, 10]. Hence, they can be used to carry out a comprehensive assessment and design a rehabilitation treatment plan [11]. However, the multitude and diversity of available tools pose a challenge in selecting the most appropriate one for evaluating the upper extremity skills of children with cerebral palsy. One method to address this challenge is to recognize, compare, and select tools based on their content, psychometric properties, method of implementation, and clinical utility [12]. In this regard, Greaves et al. [13] in 2010, as well as Elvrum et al. [14] in 2016, respectively, explored assessment tools for bilateral upper limb skills and those specific to assessing upper limb skills in children with diplegia. While these studies have been instrumental in introducing assessment tools for upper limb skills in children with cerebral palsy, none have specifically focused on suitable tools for assessing unilateral skills in this population. Consequently, many of the tools utilized in this field have not been included among the listed items due to the criteria for inclusion and exclusion of these articles. However, the assessment of unilateral upper limb skills is considered one of the common indicators in evaluating upper limb performance [15, 16]. Therefore, introducing tools that assess the skills of children with cerebral palsy in this domain can facilitate the process of comparing and selecting available tools for researchers and therapists. In this regard, one study method that has recently gained attention and is better suited to achieve this goal is the umbrella review. This type of study, by critically evaluating, summarizing, comparing, and examining the discrepancies of multiple systematic reviews in a particular field, makes decision-making easier for therapists and provides useful information on a specific topic in a short time [17]. Since the components of this study method are systematic review articles, it can be considered among the highest levels of evidence [18]. Thus, in this article, we aimed to first critically assess the quality of systematic reviews conducted on upper limb tools using this review method. Subsequently, in the next steps, we sought to identify tools that evaluate one-handed skills of children with cerebral palsy at the activity level of the ICF and to explain their psychometric properties in detail. This would provide a comprehensive set that enables practitioners in the field of pediatric rehabilitation to familiarize themselves with, compare, and select the available tools.

## 2. Methods

### 2.1. Searching and Selecting the Articles

**Figure 1:**
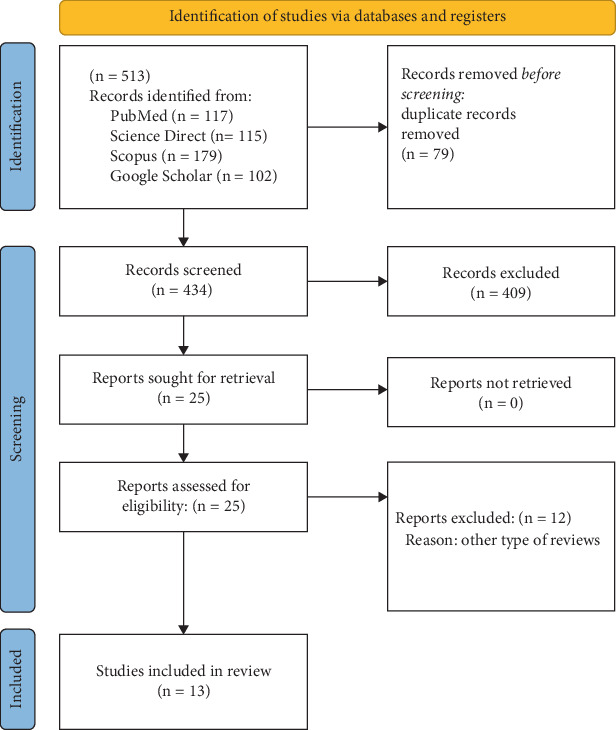
The PRISMA 2020 flow diagram.

In this umbrella review, the keywords “assessment,” “measurement,” “tool,” “evaluation,” “outcome,” “test,” “hand function,” “upper limb,” “upper extremity,” “arm,” “hand,” “wrist,” “finger,” “elbow,” “forearm,” and “cerebral palsy,” which were taken from the review of articles and medical subheadings terms, were searched using search operators in databases like Scopus, Web of Science, PubMed, and Google Scholar. Using the mentioned keywords, two researchers (S.B. and M.P.) independently examined the systematic reviews published from 2000 to 2024 and read the articles based on the title and abstract after integrating the search results in the Endnote software and removing the duplicates. At this stage, two researchers (S.B. and M.P.) independently reviewed all the articles based on the inclusion and exclusion criteria and selected the final articles for further review. The cerebral palsy community, type of systematic review, and evaluation of the upper extremity tools were the inclusion criteria for this research. The exclusion criteria, however, were articles in the non-English language and reviewing only upper extremity classification systems. Then, the quality of the selected articles was reviewed based on the JBI Critical Appraisal Checklist for Systematic Reviews and Research Syntheses [19], and all the tools introduced in these articles, including the imported and exported tools, were selected for further review. All steps are illustrated in the PRISMA diagram (Figure 1).

### 2.2. Selection of Tools and Extraction of Information

Given the variation in age ranges across different studies regarding the acquisition of one-handed skills, this review specifically focuses on children aged 3 years and older [20]. Consequently, assessment tools were selected based on their coverage of this age range and if at least 55% of their items encompassed one-handed skills. This criterion is aimed at ensuring a thorough investigation of the psychometric properties within the cerebral palsy population. Tools that solely assessed children's upper extremity function at the participation level or body function and structure of the ICF or those that did not include children with cerebral palsy in their psychometric studies were excluded. Finally, the remaining tools were independently evaluated by two researchers (S.B. and D.H.) using the CanChild Outcome Measure Rating Form, and their psychometric characteristics were reported [21]. If there was a discrepancy in scoring between the two researchers, a third evaluator (A.M.) would rescore the tools, and the final score for each tool was then recorded.

## 3. Results

### 3.1. Review of Articles and Tools

After removing the duplicates and screening, a total of 25 reviews were selected for the initial review. By reading the full text of articles, 13 articles were selected based on the inclusion and exclusion criteria for the review and extraction of upper extremity tools. The results of the review based on the JBI Critical Appraisal Checklist for Systematic Reviews and Research Syntheses are presented in Table 1, and the title of the tools identified in each article is reported in Table 2. More than half of the selected review articles lacked proper review tools for selecting the initial articles or had a poor review process conducted by two researchers. Also, ambiguity in expressing the methods used to reduce the error when extracting data and failure to provide suggestions for future studies were other weaknesses of these articles.

After examining the selected articles and collecting the tools used in each study (both included and excluded tools), 149 tools were identified for the studies related to the upper extremities of children with cerebral palsy. After checking the tools according to the inclusion and exclusion criteria, 25 tools were finally selected for the evaluation of psychometric properties. Tools such as the Purdue Pegboard Test, Wolf Motor Function Test, Grooved Pegboard Test, Annett's Peg Moving Task, Comparative Analysis of Performance-Motor, In-hand Manipulation Test, Toddler Arm Use Test, and Modified Nine-Hole Peg Test (N_HPT), despite being used in studies on children with cerebral palsy, were excluded from the profile table due to the lack of psychometric data in this population. Finally, the psychometric characteristics of 17 tools were reported in Table 3. Other descriptive features of this tool are explained in detail below.

### 3.2. Other Features of the Tools

#### 3.2.1. Quality of Upper Extremity Skills Test (QUEST)

It is a criterion-oriented and standardized questionnaire designed and printed by Dematteo et al. in 1992 [31] for children with neurological motor disorders along with spasticity, with an age range of 18 months to 8 years. This test measures the quality of the upper extremity function in children with cerebral palsy through 34 questions in four dimensions, including dissociated movement, grasp, protective extension, and weight bearing. The items of this questionnaire are designed based on the normal developmental sequence and evaluate each upper limb separately. The responses to this questionnaire are dual (yes or no) and their scores are calculated and reported based on the formula suggested in this test. In this test, a higher score indicates a better quality of upper extremity function, which is calculated as a percentage and reported separately for each part. The administration and scoring of this test take 30–45 min, and it can be obtained for free. Based on the investigations, this test is suitable for use in the Manual Ability Classification System (MACS) Levels 1–5 [32, 34, 87].

#### 3.2.2. Box and Blocks Test of Manual Dexterity (BBT)

It is a standard tool designed by Mathiowetz et al. in 1985 [35]. This time-based tool measures the gross manual dexterity of children aged 3 years and older while moving blocks from one side of the compartment to the other side [88]. This test starts with the dominant upper extremity, and the child has 60 s to transfer the blocks from one side of the box, which is separated by a middle wall, to the other side of the box. In the end, the number of blocks transferred to the opposite side is counted and recorded. This process is also repeated in the nondominant part, and the number of transferred blocks is taken into consideration [36]. The size of the blocks in this test (2.5 cm) is such that even children with weak upper limbs can move several blocks [89]. The information of this test is normalized based on the affected and healthy population. It is implemented in 10 min and does not require special training [28, 89].

#### 3.2.3. Jebson–Taylor Hand Function Test (JTHFT)

This test was first designed in 1969 by Jebson et al. and Taylor et al. to measure the effect of treatment on the hand function of adults, and after a few years, it was standardized for use in the age range 6–19 years [39, 90]. This test is normalized based on age and gender and measures the speed and dexterity of a person doing seven daily activities with one hand [89]. The seven subsets of this test include writing, turning over cards, picking up small objects, stacking checkers, simulated feeding, moving light cans, and heavier (1 lb) cans. The time of doing each of these is recorded with the dominant hand and the nondominant hand, and the sum of these times is taken as a person's functioning score. Less time means better performance. Item scores (and total scores for children) are compared with normative tables according to age and sex. Reassessments allow clients to be compared with their own scores as well as with norms to measure the effectiveness of intervention [8]. This test is performed within 15–30 min and does not require special training. The set of tools for this test can be purchased online. However, one can get information about how to make the tool by reading the main article of this test [28, 90].

#### 3.2.4. Melbourne Assessment of Unilateral Upper Limb Function (MAUUL)

It is a criterion-based tool that was designed and published in 1999 by Randall et al. to evaluate the quality of upper extremity function of children with cerebral palsy and neurological injuries aged 5–15 years [43]. It examines the upper extremity function of children with cerebral palsy through 16 items in the form of activities such as reaching, grasping, releasing, and manipulating objects. The items of this test can be administered in a standard way within 30 min. The administration of all these items is filmed according to the manual of this tool, and their scoring is done based on reviewing the recorded video, which takes approximately 30 min. Each item has its scoring system, which can include numerous sub-skills and a scoring system of 3, 4, or 5 for each subskill. Each subskill describes different characteristics such as range, accuracy, and fluency of movement [28, 91].

Additional studies conducted on this test by Randall et al. using Rasch analysis led to the introduction of the second version of this tool, called Melbourne Assessment of Unilateral Function 2 (MAUL 2) in 2012. MAUL 2 considers four unidimensional subscales, including the range of motion, fluency, accuracy, and dexterity, to define the quality of upper extremity function. Compared to the original test, it has 14 items, and the scoring of several items has been modified [48]. This test is appropriate for children with neurological disorders, from 2 years and 6 months to 15 years, and is more feasible and efficient than its previous version. Also, the possibility of clinical interpretation of its scores has increased [42, 48]. This test is rated using a 3-, 4-, or 5-point scoring system or based on a criterion defined by an individual, which requires 30 min to complete. The scores of different parts of this questionnaire can be reported separately and used in children with MACS 1-5 [14, 42].

#### 3.2.5. Pediatric Motor Activity Log (PMAL)

It is a parent-oriented tool designed by Taub et al. in 2004 [92] to determine the extent of the affected upper limbs of children with hemiplegic cerebral palsy in daily life activities, aged from 7 months to 8 years [50, 92]. This tool employs two 6-point criteria to evaluate the performance of 22 different activities and determine how often and how well the affected upper limb is used during daily activities [26]. Studies have not reported how to select the items in this questionnaire. However, this tool has been designed based on the Motor Activity Log Test, which is specific to adults with stroke [26]. PMAL is a semistructured interview whose items include unilateral functioning tasks such as turning a doorknob and pulling a toy with a rope. The investigations conducted using the Rasch analysis on the items of this test have not confirmed the construct validity and reliability of this instrument and have led to the construction of revised versions by different researchers [89].

Wallen et al. first introduced the revised version of PMAL in 2009 under the title Revised PMAL (PMAL-R). Compared to the original version, this version has 22 items on the quality of movement (how well) and 21 items on the amount of the use (how often) of the affected upper extremities and benefits from three scoring criteria. Moreover, unlike the initial version, which is a semistructured interview and is conducted by the therapist, it is a self-administered scale that is fully completed by the parent. Completing this questionnaire takes 5–15 min, and it can be used for children aged 19 months to 7 years [26, 50]. Uswatte et al.'s version is another revised version of this questionnaire that was introduced in 2012 under the title PMAL-R [52]. Unfortunately, this version is also known as the revised version of Wallen (PMAL-R). However, the two revised versions have different numbers of items, scoring criteria, and implementation methods. Therefore, when extracting information using PMAL, one should take into account sufficient accuracy about the version used [89]. PMAL-R has 22 items like the original version and uses 6-point scale. This version can be used in the age range of 2–8 years and can be conducted as a semistructured interview within 30 min [26, 52]. In any case, the psychometric properties of the two revised questionnaires have been examined and confirmed in children with cerebral palsy, but it should be kept in mind that these characteristics are specific to the revised versions and cannot be generalized to the original questionnaire [53].

#### 3.2.6. N_HPT

This is a quick and simple tool to measure fine dexterity skills. This test is a standard and normative test, which was designed by Kellor in 1971 [56] and consists of a rectangular base with a container, 9 holes (1 mm in diameter and 15 mm in depth), and 9 nails (7 mm in diameter and 32 mm in length). During the test, the client must remove nine nails from the container as quickly as possible, insert them into the holes, and then return the nails to the container one by one. The implementation time is recorded as the result of the test and is calculated separately for each hand [57, 65]. This test can be used for individuals aged 4–94 years [8].

#### 3.2.7. Shriners Hospital for Children Upper Extremity Evaluation (SHUEE)

The SHEE is a video-based assessment tool that was developed in 1996 at Shriners Children's Hospital with the aim of evaluating upper limb function in children with hemiplegic cerebral palsy. This assessment consists of two main sections, each focusing on different aspects of upper limb performance. The first section examines the active and passive range of motion of the upper limb joints, from the shoulder to the fingers, along with the degree of spasticity, assessed using the Modified Ashworth Scale. Additionally, this section evaluates the child's performance in seven selected activities of daily living and identifies treatment priorities determined by the child and their family. The second section consists of three components—spontaneous use analysis, dynamic positional analysis, and grasp-and-release analysis—which together provide a comprehensive evaluation of how the child utilizes the affected hand in different conditions. In the spontaneous use analysis component, the frequency and extent of the child's use of the affected hand during daily activities, without encouragement, are assessed using a six-point scale ranging from zero, indicating no use at all, to five, representing optimal use. The dynamic positional analysis component evaluates the alignment and control of different segments of the hand and arm, including the thumb, fingers, wrist, forearm, and elbow, during the performance of 16 specific motor tasks. Each segment is rated on a four-point scale, where zero indicates severe misalignment and three represents optimal positioning. Finally, in the grasp-and-release analysis component, the child's ability to grasp and release objects is assessed in three different wrist positions—flexed, neutral, and extended—where each position is scored between zero and one. Scoring for this assessment is based on the clinician's analysis after reviewing a recorded video of the child, providing valuable insights into the effective use of the affected hand in daily activities. Additionally, this tool facilitates monitoring functional changes over time or in response to therapeutic interventions. The final scores are expressed as a percentage of the maximum possible score. The SHEE is freely available, requires no specialized training, and can be completed in a short amount of time [58]. This evaluation can be used for children with cerebral palsy who are aged 3–18 years [30].

#### 3.2.8. Bruininks–Oseretsky Test of Motor Proficiency-2 (BOTMP-2)

This test is the second version of the BOTMP-2 and was designed and introduced in 2005 by Bruininks et al. [60] to examine the fine and gross motor skills of people aged 4–21 years. This test has 53 items that evaluate the function of people in 4 main parts, including fine manual control (with two subsets of fine motor precision and fine motor integration), manual coordination (manual dexterity and upperlimb coordination), body coordination (bilateral coordination and balance), and strength and agility (strength, running speed, and agility). In this test, the age range and the number of items have increased compared to the original test, and the implementation of some items has become easier [60]. This test is one of the most common tests used to measure the upper extremity function of children with cerebral palsy, which can be purchased and implemented based on its instructions. This test has two long and short forms, and depending on the type selected, it can be administered in 15–60 min [28].

#### 3.2.9. Peabody Developmental Motor Scales-Second Edition (PDMS-2)

This measure is the revised version of the PDMS, which was developed for children from birth up to 71 months of age. The first version of PDMS was introduced in 1983 by Rhonda Folio and Rebecca Fewell, and the second version was introduced in 2000 by the same authors [93, 94]. This version, compared to the first one, provides a wider, more accurate, and more comprehensive evaluation of motor function [63]. Like the first version, it is a norm-oriented and standardized tool that examines children's motor skills in two subsets: gross and fine motor [95]. The gross part of this tool assesses children's gross motor skills in four subsets of reflexes, stationary, locomotion, and manipulation of objects, and its upper limb part assesses children's fine motor skills in two parts: grasping and visuomotor integration, which consist of 26 and 72 items, respectively [64]. PDMS-2 is a paid tool that therapists can use individually by reading its booklet [6]. It takes 20–30 min to perform the upper extremity part of this test, and therapists measure the upper extremity skill through games and daily activities tailored to the child's age [63]. In this assessment, each item is graded on a 3-point scale of 0, 1, and 2 [96]. The raw scores of each part are summed and converted into a standard form, so it is possible to categorize the motor function into seven categories: 1: *very superior*, 2: *superior*, 3: *above average*, 4: *average*, 5: *below average*, 6: *poor*, and 7: *very poor* [97].

#### 3.2.10. Movement Assessment Battery for Children (MABC)

It is a standard and widely used test to evaluate children's motor skills. It was specifically designed in 1992 by Henderson et al. to diagnose children with mild to moderate motor disorders [69, 96]. The test includes four sets of age-related items. In each set, 8 questions evaluate the overall movement, 3 questions on upper extremity dexterity, 2 questions on ball manipulation skills, and 3 questions on static and dynamic balance. The questions on the upper extremity dexterity are scored from 0 to 5, and the dexterity set has 15 points, which can be expressed as a percentage. In this test, a lower score indicates better performance [98]. This test can be used for children aged 4–12 years [71].

#### 3.2.11. Caregivers Functional Use Survey (CFUS)

It is a standard questionnaire to measure the views of the parents of hemiplegic cerebral palsy children about the extent and manner of using the affected upper extremity in daily life activities. This survey has a different number of items depending on the version used. In 2006, Charles et al. used the 14-item version of this questionnaire, which mainly considers the functioning of the affected limb in bilateral activities [99]. However, Gordon et al. introduced the 20-item version of this questionnaire in 2007. In this version, 10 activities exclusively evaluate the one-handed skills, and the other 10 skills assess hand function in bilateral activities. Each item is rated on a 6-point Likert scale (0–5), which evaluates the extent and manner of upper extremity function of children in two parts. In this questionnaire, the average scores of unilateral or bilateral activities can be calculated and reported separately, or the combined scores of the two parts can be used together [72]. CFUS can be used for children with cerebral palsy aged 3.5 to 15.5 years [13].

#### 3.2.12. Assessment of Children's Hand Skills (ACHSs)

This tool was first designed and introduced by Chien et al. in 2010 for healthy and disabled children [74]. Based on the observation of children's performance in the real environment, the efficiency of using the upper extremities is determined in real-life situations. For this purpose, the test considers 22 activities with different levels of difficulty. These activities reflect the usual occupations of children in areas related to school and education (8 items), daily activities (6 items), and play and recreation (8 items). Then, to increase the client-oriented dimension, in a part of the questionnaire, parents' views are asked regarding the activities that can create a suitable challenge for the child as well as the suitable environment for observing these activities. Based on the parents' responses, the therapist selects 2 or 3 out of 22 activities (not all) and observes the child's performance in each activity for 5–10 min at most. During the observation, the therapist determines the efficiency of using the upper limb for each of the hand skills based on a 6-point Likert scale (1: *very inefficient* to 6: *very efficient*). This test includes 20 hand skills, which can be divided into six categories: manual gesture (1 item), body contact hand skills (1 item), adaptive skilled hand use (5 items), arm-hand use (7 items), bimanual use (3 items), and general quality (3 items). It takes 20–30 min to perform this criterion-referenced test, and its scores can be reported in three forms: raw, percentage, or converted using Face Rasch software [76, 78]. This measure can be used for children with cerebral palsy who are aged 2–12 years [76].

#### 3.2.13. Canadian Occupational Performance Measure (COPM)

It is an individual client-centered measure designed by Law et al. in 1990 [79] to detect clients' changes in “self-perception” in occupational performance. This measure examines occupational performance in three areas, including personal care, productivity, and leisure time, and is performed in five phases. In the first phase, the therapist asks the client to think about a day in their life and specify the things they need, want, or are expected to do, but are unable to do, do not do, or the way they do them does not satisfy them. Then, in the second phase, clients are asked to rate each activity according to its importance in their lives, based on a 10-point Likert scale, from 1 as the least important to 10 as the most important. In the third phase, using the obtained information, the clients are requested to choose the five most important problems. The fourth and fifth phases of this measure are done by re-evaluating the questionnaire and calculating its score. For each of these problems, the client determines the level of usual performance and the level of satisfaction with the implementation of that activity on a 10-point Likert scale from 1 as the lowest to 10 as the highest. The calculation of changes in this questionnaire is done by determining the total score, which is calculated by adding up performance or satisfaction scores for all problems and dividing them by the number of problems, and changes greater than or equal to 2 are reported as significant [8, 82, 100]. This measure needs to be purchased and takes 30–45 min to complete [86]. COPM can be used for individuals aged 7 years and older [8].

#### 3.2.14. Goal Attainment Scaling (GAS)

This criterion was first designed in 1960 by Kirusek and Sherman for use in the mental health domain. However, its modified version can be used for all ages and diagnoses. GAS is a semistructured interview with a 5-point scale (from −2: much worse than expected to +2: much better than expected) to determine the progress and access of people to their goals during the treatment period. In this scale, problem areas are identified and weighted based on their importance and difficulty and selected for treatment. The goals at this stage should be chosen in such a way that they are accessible and determined completely based on SMART principles [84]. The use of this criterion does not require special training, although it is recommended to be administered by experienced therapists [86].

## 4. Discussion

### 4.1. Quality of Articles

The present review was designed and conducted to identify and introduce the tools related to the assessment of one-handed skills among children with cerebral palsy. A total of 13 systematic reviews conducted from 2000 to 2024 to identify the upper extremity tools in children with paralysis, regardless of the type of cerebral palsy, were reviewed. In this review, more than 50% of the known systematic reviews did not evaluate the quality of the clinical trials or did not seek help from a sufficient number of researchers to review the articles. This can be a reason for the abundance of known tools in this study compared to previous studies. Failure to measure the quality of clinical trials by scales such as PEDro or other tools can be effective in the inclusion of low-quality studies in systematic reviews and can indirectly influence the inclusion of different tools with insufficient psychometric characteristics in the review articles of this field.

### 4.2. Tools

One of the key factors in selecting an assessment tool is its psychometric properties, including sufficient validity and reliability, as well as the availability of supporting evidence in these areas [30]. In this regard, the review conducted showed that the tools included in this study provided sufficient evidence of validity and reliability for use in children with cerebral palsy. However, the PMAL test lacked the necessary evidence regarding reliability for this population. This could potentially be attributed to the tool's weakness in achieving construct validity and the development of revised versions by Wallen and later by Uswatte. These revised versions, whose validity and reliability have been examined in the cerebral palsy population, exhibit better psychometric characteristics for this group.

Additionally, the results of this review indicated that the PMA_R version by Uswatte, alongside the MUUL and PDMS-2 tools, provided the most comprehensive psychometric information for assessing children with cerebral palsy, with all different psychometric aspects having been examined in at least one study involving this population. More detailed evaluations of the tools' validity, based on the Can Child Outcome Measure Rating Form, showed that in terms of content validity, the first and second versions of MUUL, QUEST, PDMS-2, and PMAL_R (Wallen version) performed excellently. Regarding criterion validity, the PDMS-2 and ACHS tools achieved excellent levels of evidence. Reliability assessments revealed that only the QUEST tool demonstrated excellent evidence for interrater and intrarater reliability, while the PMAL_R (Uswatte version) showed excellent evidence for internal consistency.

In terms of overall clinical utility, aside from adequate psychometric properties, various factors may influence the choice of tool, including the cost of obtaining the tool, the need for training, accessibility, and the time required to administer the test [31]. Based on the Can Child Outcome Measure Rating Form, the PMAL_R (Uswatte version), COPM, and ACHES tools earned excellent scores and were considered most suitable for clinical use.

## 5. Conclusion

The current study was an attempt to identify and introduce the tools utilized for assessing one-handed skills in children with cerebral palsy through a comprehensive review of systematic reviews focused on upper limb assessments. Furthermore, the quality of the studies was examined as a secondary goal of the research. These studies showed that more than half of the systematic reviews conducted in this field did not evaluate the quality of the implementation of primary clinical trials. Although it may not be important in reviews to identify the tools and review the quality of clinical trials the same as the reviews conducted on specific interventions, the selection of articles with acceptable quality can help identify valid tools faster. Therefore, it is recommended that future systematic review studies in the field of tool identification place greater emphasis on the quality of clinical trial articles. Additionally, through the review of the literature, 18 tools with psychometric properties for assessing one-handed skills in children with cerebral palsy were identified. Among these, considering both psychometric properties and clinical utility factors such as administration time, training requirements, accessibility, and test procurement, the PMAL_R (Uswatte version), COPM, and ACHES tools received excellent scores for clinical use.

## Figures and Tables

**Table 1:** The result of Johanna Briggs Institute critical appraisal checklist criteria for selected systematic review (*N*:13).

	**Percentage%**			
	**Yes**	**No**	**Unclear**	**Not applicable**
1. Is the review question clearly and explicitly stated?	100			
2. Were the inclusion criteria appropriate for the review question?	84.6		15.4	
3. Was the search strategy appropriate?	92.3		7.7	
4. Were the sources and resources used to search for studies adequate?	100			
5. Were the criteria for appraising studies appropriate?	46.2	53.8		
6. Was critical appraisal conducted by two or more reviewers independently?	23.1	53.8	15.4	7.7
7. Were there methods to minimize errors in data extraction?	53.8	7.7	38.5	
8. Were the methods used to combine studies appropriate?	69.2	7.7	23.1	
9. Was the likelihood of publication bias assessed?				100
10. Were recommendations for policy and/or practice supported by the reported data?	92.3		7.7	
11. Were the specific directives for new research appropriate?	61.5	7.7	30.8	
12. Overall appraisal: Include/exclude/seek further info	100			

**Table 2:** Descriptive analysis of selected systematic reviews of assessment tools of upper limb function of children with cerebral palsy (*n* = 13).

**Authors/year**	**Title**	**AAT/RAT**	**Reviewed assessment tools**	**Measurement properties**	**Conclusion**
Thomé Teixeira da Silva et al. (2022) [22]	Selecting assessment tools to characterize upper limb function of children with cerebral palsy: A mega-review of systematic reviews	84/14	• ABILHAND-Kids • Assisting Hand Assessment (AHA) • Children's Assessment of Participation and Enjoyment/Preferences for Activities of Children (CAPE/PAC) • Children's Hand-Use Experience Questionnaire (CHEQ) • Canadian Occupational Performance Measure (COPM) • Children Participation Questionnaire (CPQ) • Melbourne Assessment of Unilateral Upper Limb Function (MUUL) • School Functional Assessment– Participation section (SFA-P) • Both Hands Assessment (BoHA) • Hand Assessment for Infants (HAI) • Mini-Assisting Hand Assessment (Mini-AHA) • Melbourne Assessment of Unilateral Upper Limb Function -2 (MUUL-2) • Pediatric Motor Activity Log-revised (PMAL-R) • Quality of Upper Limb Extremity Skills Test (QUEST)	• Reliability ✓ Internal consistency ✓ Test–retest ✓ Intrarater ✓ Interrater • Validity ✓ Content ✓ Construct ✓ Convergent ✓ Structural ✓ Cross-cultural ✓ Responsiveness	• The ABILHAND-Kids • Assisting Hand Assessment (AHA) • Melbourne Assessment of Unilateral Upper Limb Function (MUUL)

Kalle et al. (2022) [23]	Quality of patient- and proxy-reported outcomes for children with impairment of the upper extremity: a systematic review using the COSMIN methodology	97/13	• ABILHAND-Kids • Children's Arm Rehabilitation Measure (ChARM) • Children's Hand-use Experience Questionnaire (CHEQ) • Child Health Questionnaire (CHQ) • Children's Hand-Skills ability Questionnaire (CHSQ) • Duruöz Hand Index (DHI) • Hand-Use-at-Home Questionnaire (HUH) • Infant Motor Activity Log (IMAL) • Pediatric Evaluation of Disability Inventory (PEDI) • Pediatric Outcomes Data Collection Instrument (PODCI) • Patient-Reported Outcomes Measurement Information System, computer-adaptive test (PROMIS,CAT) • Disabilities of the Arm, Shoulder and Hand (DASH) Pediatric Motor Activity Log(PMAL)	• Reliability ✓ Internal consistency ✓ Test–retest ✓ Intrarater ✓ Interrater • Validity ✓ Content ✓ Construct ✓ Convergent ✓ Structural ✓ Cross-cultural ✓ Responsiveness	No specific suggestion

Burgess et al. (2019) [6]	A systematic review of upper limb activity measure for 5 to 18 children with bilateral cerebral palsy	48/8	• Melbourne Assessment of Unilateral Upper Limb Function (MUUL) • Melbourne Assessment of Unilateral Upper Limb Function 2 (MUUL_2) • Peabody Developmental Motor Scale 2 – Fine motor (PDMS) • Both Hands Assessment (BoHA) • ABILHAND-Kids • ACTIVLIM-CP • Children's Arm Rehabilitation Measure (ChARM) • Patient-Reported Outcomes Measurement Information System, Pediatric Upper Limb - short form (PROMIS-PUL)	• Reliability ✓ Internal consistency ✓ Measurement error • Validity ✓ Cross-cultural ✓ Criterion ✓ Construct ✓ Structural ✓ Content → Relevance → Comprehensibility → Comprehensiveness ✓ Responsiveness	• ACTIVLIM-CP • Both Hands Assessment (BoHA)

Elvrum et al. (2016) [14]	Outcome measures evaluation hand function in children with bilateral cerebral palsy: a systematic review	41/6	• ABILHAND-Kids • Erhardt Developmental Prehension Assessment (EDPA) • Peabody Developmental Motor Scales (PDMS) • Quality of Upper Extremity Skills Test (QUEST) • Melbourne Assessment of Unilateral Upper Limb Function (MUUL) Melbourne Assessment of Unilateral Upper Limb Function-2 (MUUL_2)	• Reliability ✓ Internal consistency ✓ Intrarater ✓ Interrater ✓ Test–retest ✓ Measurement error • Validity ✓ Construct→hypotheses testing ✓ Content ✓ Structural validity ✓ Criterion ✓ Responsiveness	• ABILHAND-Kids • Melbourne Assessment of Unilateral Upper Limb Function-2 (MUUL_2)

Santos et al. (2015) [24]	Upper limb function evaluation scale for individuals with cerebral palsy: a systematic review	NR/8	• Pebody developmental motor scale (PDMS) • Pebody developmental motor scale-2 (PDMS-2) • Quality of Upper Extremity Skills Test (QUEST) • Melbourne Assessment of Unilateral Upper Limb Function (MUUL) • Bruninks–Oseretsky test of motor proficiency (BOTMP) • Pediatric motor activity log (PMAL) • Besta scale • Pediatric quality of life (PQOL)	Not Reported	No specific suggestion

Krumlinde-Sundholm et al. (2015) [25]	What assessments evaluate use of hands in infants? A literature review	10/4	• Bayley Scale of Infant and Toddler Development–Version III (BSID-III) • Grasping and Reaching Assessment of Brisbane (GRAB) • Peabody Developmental Motor Scales–Version 2 (PDMS-2) • Toddler and Infant Motor Evaluation (TIME) • Hand Assessment for Infants (HAI) • Infant Motor Profile (IMP) • Movement Assessment of Infants (MAI) • Posture and Fine Motor Assessment of Infants (PFMAI)	• Validity • Reliability	• Grasping and Reaching Assessment of Brisbane (GRAB) • Hand Assessment for Infants (HAI)

Wallen and Stewart (2015) [26]	Upper limb function in everyday life of children with cerebral palsy: description and review of parent report measures	NR/6	• The ABILHAND-Kids • Children's Hand-Use Experience Questionnaire (CHEQ) • Caregivers Functional Use Survey(CFUS) • Original and revised versions of the pediatric motor activity log (PMAL) • Infant motor activity log (IMAL)	• Reliability ✓ Test–retest ✓ Stability ✓ Internal consistency • Validity ✓ Construct ✓ Content ✓ Discriminative ✓ Convergent ✓ Sensitivity to change	• The ABILHAND-Kids • Revised Pediatric Motor Activity Log (PMAL-R) • Children's Hand-use Experience Questionnaire (CHEQ)

Chien et al. (2014) [27]	Measures of participation outcomes related to hand use for 2- to 12-year-old children with disabilities: a systematic review	65/9	• The Children Helping Out: Responsibilities, Expectations, and Supports (CHORES) • The Paediatric Activity Card Sort (PACS) • The Paediatric Interest Profile (PIP) • The Children's Assessment of Participation and Enjoyment/Preferences for Activities of Children (CAPE/PAC) • The School Function Assessment-Participation section (SFA-P) • The Children Participation Questionnaire (CPQ) • The Children's Leisure Assessment Scale (CLASS) • The Preschool Activity Card Sort (Preschool ACS) Assessment of Life Habits (LIFE-H)	• Reliability ✓ Internal consistency ✓ Test–retest ✓ Intrarater ✓ Interrater • Validity ✓ Content ✓ Construct ✓ Criterion ✓ Responsiveness	• Children's Assessment of Participation and Enjoyment/Preferences for Activities of Children (CAPE/PAC) • The School Function Assessment-Participation section (SFA-P) • The Children Participation Questionnaire (CPQ)

Lemmens et al. (2012) [9]	Valid and reliable instruments for arm-hand assessment at ICF activity level in persons with hemiplegia: a systematic review	NR/8	• Quality of Upper Limb Extremity Skills Test (QUEST) • Shriners Hospital for Children Upper Extremity Evaluation(SHUEE) • The ABILHAND-Kids • Canadian Occupational Performance Measure (COPM) • Goal Attainment Scaling(GAS) • Pediatric Motor Activity Log (pMAL) • Upper extremity item bank (UE Item Bank) • Assisting Hand Assessment (AHA) • Actual Amount of Use Test (AAUT) • Activities of Daily Living observation (ADL observation) • Arm Motor Ability Test (AMAT) • Assessment of Motor and Process Skills (AMPS) • Action Research Arm Test (ARAT) • Chedoke Arm Hand Actvity Inventory (CAHAI) • Frenchay Arm Test (FAT) • Functional Test for the Hemiplegic Upper Extremity (FTHUE) • Jebsen–Taylor Hand Function Test (JTHFT) • Lindmark Motor Assessment Scale (LMAS) • Melbourne Assessment of Unilateral Upper Limb Function (MUUL) • Motor Evaluation Scale for Upper Extremity Stroke Patients (MESUPES) • Manual Function Test (MFT) • Upper Extremity Performance Test for Elderly (Test d'Evaluation des Membres supérieurs de Personnes Agées) (TEMPA) • Upper Body Dressing Scale (UBDS) • Video Observation Aarts and Aarts – DDD (VOAA-DDD) • Wolf Motor Function Test (WMFT) • Duruoz Hand Index (DHI) • Hand Function Survey (HFS) • Motor Activity Log (MAL) Functional Arm Activity Behavioral Observation System (FAABOS)	• Reliability • Validity ✓ Responsiveness	No specific suggestion

Wagner and Davids (2012) [28]	Assessment Tools and Classification Systems Used For the Upper Extremity in Children With Cerebral Palsy	NR/21	• Assisting Hand Assessment (AHA) • Box and Block test (BBT) • House Functional Classification System (House) • Jebsen Taylor Hand Function Test (JTHF) • Melbourne Assessment of Unilateral Upper Limb Function (MUUL) • Quality of Upper Extremity Skills Test (QUEST) • Shriners Hospitals Upper Extremity Evaluation (SHUEE) • Evaluation of Disability Inventory (EDI) • Functional independence measure (WeeFim) • ABILHAND-Kids • Activities Scale for Kids (ASK) • Cerebral Palsy Quality of Life (CP-QOL) • Children's Assessment of Participation and Enjoyment/Preferences for Activities of Children (CAPE/PAC) • Children's Hand-use Experience Questionnaire (CHEQ) • Child Health Questionnaire (CHQ) • Life Habits Assessment (LHA) • Manual Ability Classification System (MACS) • Pediatric Quality of Life Inventory–Cerebral Palsy Module (PQOL-CP) • Canadian Occupational Performance Measure (COPM) • Pediatric Outcomes Data Collection Instrument (PODCI) Goal Attainment Scaling (GAS)	• Reliability • Validity	No specific suggestion

Klingels et al. (2010) [29]	A systematic review of arm activity measures for children with hemiplegic cerebral palsy	18/11	• Quality of Upper Extremity Skills Test (QUEST) • Melbourne Assessment of Unilateral Upper Limb Function (MUUL) • Shriners Hospital Upper Extremity Evaluation (SHUEE) • Assisting Hand Assessment (AHA) • Video Observations Aarts and Aarts (VOAA) • Bruininks–Oseretsky Test of Motor Proficiency (Subtest 8) (BOTMP) • Peabody Developmental Motor Scales–Fine Motor Abilities (PDMS) • Revised Pediatric Motor Acivity Log (PAML_R) • Jebsen Taylor Hand Function Test (JTHF) • ABILHAND-Kids Pediatric Evaluation of Disability Inventory (PEDI)	• Validity ✓ Construct ✓ Content ✓ Concurrent • Reliability ✓ Intrarater ✓ Interrater ✓ Test–retest ✓ Standard Error of Measurement (SEM) ✓ Smallest detectable difference (SDD)	• ABILHAND-Kids • Revised Pediatric motor activity log (PMAL_R) • Pediatric evaluation of disability inventory (PEDI)

Greaves et al. (2010) [13]	Assessing bimanual performance in young children with hemiplegic cerebral palsy: a systematic review	NR/11	• Assisting Hand Assessment (AHA) • Caregivers Functional Use Survey (CFUS) • House Functional Classification System (House) • Goldner • Pagliano et al. • Satila et al. • Fedrizzi et al. • Crocker et al. • Dickerson and Brown • Smelt et al. • Fergus et al.	• Reliability • Validity ✓ Content validity ✓ Construct validity	• Assisting Hand Assessment (AHA)

Gilmore et al. (2010) [30]	Upper limb activity measures for 5- to 16-year-old children with congenital hemiplegia: a systematic review	36/5	• ABILHAND-Kids • Assisting Hand Assessment (AHA) • Melbourne Assessment of Unilateral Upper Limb Function (MUUL) • Quality of Upper Extremity Skills Test (QUEST) • Shriners Hospital Upper Extremity Evaluation (SHUEE)	• Validity ✓ Content ✓ Construct ✓ Criterion ✓ Evaluative ✓ smallest detectable difference • Reliability ✓ Internal consistency ✓ Interrater ✓ Intrarater ✓ Test–retest	• Melbourne Assessment of Unilateral Upper Limb Function (MUUL) • Assisting Hand Assessment (AHA) • ABILHAND-Kids

Abbreviations: AATs: achieved assessment tools, NR: not reported, RATs: reviewed assessment tools.

**Table 3:** Measurements properties of assessment tools of unilateral upper limb function of children with cerebral palsy (*n* = 18).

**Assessment tools**	**Measurement properties**								**Overall utility**
	**Validity**				**Reliability**				
	**Content**	**Construct**	**Criterion**	**Responsiveness**	**Internal consistency**	**Test–retest**	**Intrarater**	**Interrater**	
QUEST Dematteo et al. (1992) [31]	Literature review, discussion with clinicians and experts [30]	Pearson product moment correlation coefficient between QUEST total score and left and right-hand function rating were 0/72 and 0/58 Pearson product moment correlation coefficient between chronological age and the quest total score was 0/33. [31]	High correlation between QUEST total score and PDMS-FM (Pearson product moment correlation coefficients: 0/84) [31]	—	*α*: 0.97 [32]	ICC: 0/75–0/95 [31] Spearman correlation coefficients for test total: 0/92 (range: 0/84–0/94) [33]	Spearman correlation coefficient: 0/63–0/95 [33] ICC: 0/69/–0/89 [34] ICC total: 0.96 (range: 0.93–0.98) [32]	ICC: 0/51–0/96 [31] Spearman correlation coefficient: 0/72–0/90 [33] ICC total: 0.91 (range: 0.80–0.96) [34] ICC total: 0.86 (range: 0.73–0.93) [32]	Clinical utility: Excellent easily available: Yes (^+^) RSS Validity: Adequate RSS Reliability: Excellent
CanChild Outcome Measure Rating	Excellent	Adequate	Adequate	No evidence available	Adequate	Adequate	Excellent	Excellent	Adequate
BBT Mathiowetz (1985) [35]	The different normalizations and their applications across various age groups and populations confirm the content validity of this test [35, 36]. However, no data is available on how this tool was developed.	The score of the BBT had moderate to strong correlations s between BBT and four subtests of the MUUL_2 (*r*s: 0.63–0.78) [37] moderate correlations between the subtest 3 of the BOTMP-2 (*r*s: 0.49–0.57) [37] moderate correlations between BBT and the amount of use/quality of movement of the PMAL-R (*r*s: 0.51–0.63) [37]	—	Clinical significant difference was 1.9 (blocks) on the more affected hand and 3.0 (blocks) on the less affected hand [38] The unilateral cp group showed significant changes for the more affected hand (*p* < 0.001) The effect size showed moderate changes in both hands (more affected hand, effect size = 0.678; less affected hand, effect = 0.514) the bilateral cp group, showed significant changes on the more affected hand (*p* = 0.010) and a clear trend on the less affected hand (*p* = 0.052). and the effect size showed large changes for the more affected hand (effect size = 0.936) and moderate changes in the less affected hand (effect size =0.496) [38]		ICC > 0.849 [38] ICC: 0.98 [37]	—	—	Clinical utility: Excellent easily available: Yes (^a^) RSS Validity: Adequate RSS Reliability: Adequate
CanChild Outcome Measure Rating	Adequate	Adequate	No evidence available	Adequate	No evidence available	Adequate	No evidence available	No evidence available	Adequate
JTHFT Jebsen-Taylor (1969) [39]	The content validity of this tool has been examined through comparison with other assessment tools and its use in various studies [40] However, no data is available on how this tool was developed.	Significant positive Pearson's correlation coefficients between the Italian version of the JTHFT and the MACS results for both the dominant hand (value between: 0.557–0.738) and the nondominant hand (value between: 0.529–0.724) [41]	—	Minimal clinically important difference was 54.7 s on the more affected hand and 20.9 s on the less affected hand [38] Significant changes on the more affected hand of unilateral cp (*p* < 0.001). The effect size showed small change in the more affected hand (effect size = 0.489) [38] significant changes on the more affected hand (*p* = 0.005). The effect size showed moderate changes on the more affected hand (effect size = 0.594) [38]	Nondominant hand *α*: 0.944 [41] Dominant hand *α*: 0.911 [41]	ICC > 0.932 [38]	—	—	Clinical utility: Adequate easily available: Yes (^a^) RSS Validity: Adequate RSS Reliability: Adequate
CanChild Outcome Measure Rating	Adequate	Adequate	No evidence available	Adequate	Adequate	Adequate	No evidence available	No evidence available	Adequate
MUUL Randall et al. (1999) [42]	Literature review, review of existing assessments, workshops with experienced occupational therapist [30]	Very strong correlation coefficients were calculated between the Melbourne Assessment and self-care (Spearman's rho correlations:0.939) strong correlation coefficients were calculated between the Melbourne Assessment and mobility domains (Spearman's rho correlations:0.783) of the PEDI and the overall functional skills section of the PEDI (r:0.718) [43]	Good correlation between the score of Melbourne and the score of BBT (Pearson's correlation coefficients = 0.816) [44]	The smallest detectable difference was 3.2% and 8.9% [45]	*α* = 0.96 [46]	For test totals: ICC: 0.98 and 0.97 [46]	ICC: (0.97) [46]	ICC: (0.95) [46] ICC: (0.961) [47]	Clinical utility: Adequate easily available: Yes (^a^) RSS Validity: Excellent RSS Reliability: Adequate
CanChild Outcome Measure Rating	Excellent	Adequate	Adequate	Adequate	Adequate	Adequate	Adequate	Adequate	Adequate
MUUL-2 Randall et al. (2012) [48]	Further investigation of the original Melbourne Assessment AND use statistical method (Rasch analysis)	—	—	Ranging from 13.6% to 20.9% across subscales for test–retest reliability of the more affected upper limb [49]	—	ICC: 0.94-0.97 [49]	ICC: 0.97–1.00 [49]	ICC: 0.930.99 – [49]	Clinical utility: Adequate easily available: Yes (^a^) RSS Validity: Adequate RSS Reliability: Adequate
CanChild Outcome Measure Rating	Excellent	No evidence available	No evidence available	Adequate	No evidence available	Adequate	Adequate	Adequate	Adequate
PMAL Tau (2004)	The PMAL is based on the Motor Activity Log; however, no report is available on the process and stages of its adaptation and development [50].	—	Significant and fair Correlation coefficients between the PMAL & the WeeFIM, PDMS-2 grasping, and PDMS- Visual-motor integration (r = 0.32–0.48) [51]	SRM: (0.89–0.99) [51] MDC for the PMAL- amount of hand use:0.67 MDC for The PMAL- quality of hand use: 0.66 for the [51]	—	—	—	—	Clinical utility: Adequate easily available: Yes (^+^) RSS Validity: Adequate RSS Reliability: Poor
CanChild Outcome Measure Rating	Adequate	No evidence available	Poor	Adequate	No evidence available	No evidence available	No evidence available	No evidence available	Poor
PMAL_R Uswatte (2012) [52]	Further investigation of the original PMAL [53]	Moderate Pearson's correlation coefficient (*r* = 0.36) between PMAL-R and PAFT limb preference scale Strong correlation of change in scores over time between the PMAL-R and PAFT (*r* = 0.5). [52]	-PMAL-R scores were strongly correlated with ABILHAND-Kids scores (How Well scale: *r* = 0.78, *p* < 0.001; How Often scale: *r* = 0.59, *p* < 0.001) [54]	MDC:0.42 [53].	*α*: 0.93 [52] *α*; How Often = 0.96, How Well = 0.97 [54] alpha = 0.97 for the HO subscale and 0.98 for the HW subscale) [55]	r: 0.89 [52] ICC: How Often = 0.98, How Well = 0.99 [54]	ICC = 0.97–0.98 [55]	ICC: 0.98–0.99 [55]	Clinical utility: Adequate easily available: Yes RSS Validity: Excellent RSS Reliability: Excellent
CanChild Outcome Measure Rating	Adequate	Adequate	Adequate	Adequate	Excellent	Adequate	Adequate	Adequate	Excellent
PMAL-R Wallen (2009) [50]	Utilizing statistical analysis (Rasch analysis) on original PMAL [50]	—		—	—	How often ICC: 0.94 [53] How well ICC: 0.93 [53]	—	—	Clinical utility: Adequate easily available: No RSS Validity: Adequate RSS Reliability: Adequate
CanChild Outcome Measure Rating	Excellent	No evidence available	No evidence available	No evidence available	No evidence available	Adequate	No evidence available	No evidence available	Adequate
N_HPT Kellor (1971) [56]	It is a standard and normative test [57]. However, there is no specific data available about the procedure of content validity.	—	—	4 and 12 s for the nonaffected and affected side [57]	—	—	Nonaffected side (ICC = 0.94) the affected side (ICC = 0.96) [57]	—	Clinical utility: Adequate easily available: Yes (^a^) RSS Validity: Adequate RSS Reliability: Adequate
CanChild Outcome Measure Rating	No evidence available	No evidence available	No evidence available	Adequate	No evidence available	No evidence available	Adequate	No evidence available	Adequate
SHUEE Shriners Hospital (1996) [58]	No evidence available [30]	Was determined through analysis of SHUEE scores for eighteen children before and after flexor carpi ulnaris to extensor carpi radialis brevis tendon transfer. SHUEE improved for all eighteen subjects & mean improvement was significant (Paired *T* Test: *p* < 0.001) Spontaneous Functional Analysis showed a significant correlation of *r* = 0.65 with the evaluated scales, while Dynamic Positional Analysis demonstrated a higher correlation of *r* = 0.85 [58] The SHUEE has strong convergent validity with PEDI (r = 0.68, p = 0.001), MACS (r = -0.68, p = 0.001), and PMAL (r = 0.86, p = 0.001) [59]	Fair correlation with the self-care scaled score from the PEDI (Pearson correlation coefficients: 0.47) [58] good inverse correlation with the nondominant total time section of the JTHFT (Pearson correlation coefficients: −0.76) [30, 58]	Sensitivity to changes calculated in five subjects who underwent treatment with botulinum toxin Type A and physical therapy, with a significant difference between pre and posttreatment evaluations in the spontaneous functional analysis (*p*: 0.02) and dynamic positional analysis (*p*: 0.01). [59]	*α*: 0.887 [59]	—	Spontaneous functional analysis (Pearson correlation coefficient: 0.99) [58] dynamic positional analysis (Pearson correlation coefficient: 0.98) [58]	Spontaneous functional analysis (ICC:0.90) [58] dynamic positional analysis (ICC = 0.89) [58]	Clinical utility: Excellent easily available: Yes (^+^) RSS Validity: Excellent RSS Reliability: Adequate
CanChild Outcome Measure Rating	No evidence available	Adequate	Adequate	Adequate	Adequate	No evidence available	Adequate	Adequate	Adequate
BOTMP-2 (upper limb subscales) Bruininks et al. (2005) [60]	This battery was developed according to the first version. It is a standard test [8]. But there is no specific data available for content validity assessment	—	Inverse correlation between the BOTMP2-UL results and the MACS level (*ρ*: −0.81, *p* value: 0.001) [61]	MDC value is 3.6755 and MCID value is 0.925 for manual dexterity subscale [62]	*α* = 0.94 [61]	—	ICC = 0.99 [61]	ICC = 0.99 [61]	Clinical utility: Adequate easily available: Yes (^a^) RSS Validity: Adequate RSS Reliability: Adequate
CanChild Outcome Measure Rating	No evidence available	No evidence available	Adequate	Adequate	Adequate	No evidence available	Adequate	Adequate	Adequate
PDMS-2 Folio and Fewell (2000) [63]	The content validity assessment was conducted through a panel of experts and health-related professionals [64]	Positive and significant correlations between subscale of PDMS-2 and age (*r*: 0.81 to 0.99) [64]	Moderate positive correlation between AIMS at 8 months and PDMS-2 at 18 months & 3 years (*r*: 0.591 and *r*:0.528) [65] Moderate Correlation Between PDMS-2 fine motor quotient and Gesell's fine motor developmental quotient (Spearman rank correlation coefficients: 0.87) [66] Correlation Between PDMS-2 fine motor quotient and Bayley-III Composite (Pearson product moment correlation coefficient: 0/89) [67]	Responsiveness coefficients: ranged from 1.7 to 2.3 [68]	McDonald's *ω*0.87: Cronbach's *α*: 0.68 Gutmann's *λ*6: 0.78 [64]	ICC: 0.88–1.00 [68] *r*: 0.99 [64]	(visual–motor integration) ICC: 0.98 [64] (grasping) ICC: 0.92 [64]	(visual-motor integration) ICC: 1.0 [64] (grasping) ICC: 0.98 [64]	Clinical utility: Adequate easily available: Yes (^a^) RSS Validity: Excellent RSS Reliability: Adequate
CanChild Outcome Measure Rating	Excellent	Adequate	Excellent	Adequate	Adequate	Adequate	Adequate	Adequate	Adequate
MABC (upper limb) Henderson and Sugden [69]	This battery was developed for DCD children based on the Test of Motor Impairment (TOMI) [70], but there is no more evidence about it.	Construct validity was examined by assessing the ability of qualitative observations to distinguish children with motor impairments (DCD and mild CP) from typically developing peers. A significant difference in total observed movement deviations (*p* = 0.007) supported the validity of these observations. [71]	—	—	—	—	—	ICC:0.75 [71]	Clinical utility: Adequate easily available: Yes (^a^) RSS Validity: Adequate RSS Reliability: Adequate
CanChild Outcome Measure Rating	Adequate	Adequate	No evidence available	No evidence available	No evidence available	No evidence available	No evidence available	Adequate	Adequate
CFUS Gordon [72]	The method used for content validity is not explained [26]	The Confirmatory Factor Analysis Index values were 0.958, and 0.976 for the Unimanual amount of use and quality of movement subscales [73]	Negative correlation between all the subscales of the CFUS and the MACS and the JTT (*p* < 0.05). [73]	—	*α*: 0.92–0.94 [73]	ICC: 0.90–0.93 [73]	—	—	Clinical utility: Poor easily available: No RSS Validity: Adequate RSS Reliability: Adequate
CanChild Outcome Measure Rating	No evidence available	Adequate	Adequate		Adequate	Adequate			Poor
ACHS Chien 2010 [74]	The ACHS demonstrated adequate content validity [74]	There were strong positive relationships between ACHS-TR and all domains of SHUEE (*r* = 0.86). Divergent validity of ACHS: (*p* = 0.70 [75] Moderate to high correlations (0.59 _ Spearman's *ρ* coefficients _ 0.89) were found with the assessments of daily living and fine motor skills [76] The ACHS correlated moderately to highly with the daily living skills questionnaire and demonstrated a varied range of correlations with the three related instruments [77] the ACHS shows preliminary evidence of construct validity for its clinical use in assessing children's hand skill performance in real-life contexts [78].	—	—	For domain 1 = 0.99 [75] *α* for domain 2 = 0.95 [75] *α* for domain 3 = 0.71 [75] *α* for domain 4 = 0.89 [75] *α* for domain 5 = 0.94 [75]	ICC = 0.99 [75] 0.42 ≤ *κ* ≤ 0.79 and the total scale level (Spearman's *ρ* = 0.78, *p* < 0.01) [74]	ICC: 0.61−0.93 [76]	ICC: 0.81 [76] Moderate interrater agreement of the total scale scores was (*ρ* = 0.63, *p* < 0.01) [74]	Clinical utility: easily available: Yes (^+^) RSS Validity: Excellent RSS Reliability: Excellent
CanChild Outcome Measure Rating	Adequate	Excellent	No evidence available	No evidence available	Adequate	Adequate	Adequate	Adequate	Excellent
COPM Law 1991 [79]	The content validity was approved based on expert occupational therapist judgments [79].	The construct validity was studied by comparing the results of the COPM with the PEDI, and a quality of life Questionnaire. results support the construct and criterion Validity [80]	The criterion validity was verified with an open-ended question results support the criterion validity [80]	COPM(Performance) coding scale can detect medium effect sizes (*p*: 0.011 effect size:0.78) [81] COPM(Satisfaction) coding scale can detect medium effect sizes (*p*: 0.019 effect size: 0.69) [81]	—	For performance *r*: 0.84 [82] for satisfaction *r* = 0.87 [82] for performance-ICC = 0.83 [83] for satisfaction- ICC scores = 0.91 [(83])	—	The interrater agreement of the prioritized problems is good enough for client-centered occupational therapy Of the prioritized problems identified in the first interview, 80% were also prioritized in the second interview [80] performance-ICC score = 0.84 satisfaction-ICC scores 0.80 [83]	Clinical utility: Adequate easily available: Yes (^a^) RSS Validity: Excellent RSS Reliability: Excellent
CanChild Outcome Measure Rating	Excellent	Adequate	Adequate	Adequate	No evidence available	Adequate	No evidence available	Adequate	Excellent
GAS Kirusek and Sherman 1960 [84]	No evidence available	—	Inconclusive correlations between GAS and PEDI change scores (Spearman's rho correlations: 0.28–0.64). [85]	The GAS Likert coding scale can detect a large effect size (*p*:0.003–effect size: 0.91) [81] The GAS Weighted coding scale can detect medium effect sizes (*p*: 0.036 effect size: 0.55) [81]	—	—	ICC = 0.96 [86]	0.51–0.95 [86]	Clinical utility: Excellent easily available: Yes (^+^) RSS Validity: Adequate RSS Reliability: Adequate
CanChild Outcome Measure Rating	No evidence available	No evidence available	Poor	Adequate	No evidence available	No evidence available	Adequate	Adequate	Adequate

*Note:K*, Kendall's coefficient (*K*).

Abbreviations: ACHS, Assessment of Children's Hand Skills; AIMS, Alberta Infant Motor Scale; BBT, Box and Block test; BOTMP-2, Bruininks–Oseretsky Test of Motor Proficiency-2; CFUS, Caregivers Functional Use Survey; COPM, Canadian Occupational Performance Measure; GAS, Goal Attainment Scaling; ICC, intraclass correlation coefficients; JTHFT, Jebsen–Taylor Hand Function Test; MABC, Movement Assessment Battery for Children; MACS, Manual Ability Classification System; MDC, minimal detectable change; MUUL, Melbourne Assessment of Unilateral Upper Limb Function; MUUL_2, Melbourne Assessment of Unilateral Upper Limb Function-2; N_HPT, Nine-Hole Peg Test; PAFT, Pediatric Arm Function Test; PDMS-FM, Peabody Developmental Motor Scales–Fine Motor; PEDI, Pediatric Evaluation of Disability Inventory; PMAL, Pediatric Motor Activity Log; PMAL-R, Pediatric Motor Activity Log-Revised; QUEST, Quality of Upper Extremity Skills Test; RSS, rigor of standardization studies; SHUEE, Shriners Hospitals Upper Extremity Evaluation; SRM, standardized response mean.

^a^Available with cost.

^+^Free available.

## Data Availability

Data sharing is not applicable to this article as no new data were created or analyzed in this study.
